# Level and Source of Copper Affects Gene Expression of Copper-Regulatory Proteins and Soluble and Mucosal Copper Concentrations in the Small Intestine of Weanling Pigs

**DOI:** 10.3390/ani16131940

**Published:** 2026-06-23

**Authors:** Robert Scott Fry, Melissa S. Ashwell, William L. Flowers, Kara R. Stewart, Karen E. Lloyd, Jerry W. Spears

**Affiliations:** 1 Department of Animal Science, North Carolina State University, Raleigh, NC 27695, USA; 2 Phibro Animal Health Corp., Teaneck, NJ 07666-6712, USA; 3 Smithfield Foods, Rose Hill, NC 28458, USA

**Keywords:** copper, Cu regulatory proteins, weanling pigs, intestinal Cu concentrations

## Abstract

A study was conducted to determine the effects of the level and source of copper (Cu) on Cu concentrations and the mRNA expression of Cu regulatory proteins in the small intestine of pigs. Thirty weanling castrated male pigs, approximately 21 days of age, were stratified by weight and assigned to dietary treatments consisting of control (6.7 mg Cu/kg from feed ingredients; no supplemental Cu) or 225 mg supplemental Cu from either Cu sulfate (CuSO_4_) or tribasic Cu chloride (TBCC). Pigs were harvested on days 35 or 36. The soluble Cu and mucosal Cu concentrations in the duodenum, proximal jejunum, and ileum were higher (*p* < 0.001) in Cu-supplemented pigs versus controls. Duodenal soluble and mucosal Cu concentrations were higher in (*p* < 0.05) CuSO_4_ versus TBCC-supplemented pigs. However, proximal jejunum and ileum mucosal Cu were higher (*p* < 0.05) in TBCC versus CuSO_4_ pigs. The intestinal copper transporter 1 (*CTR1*) expression was lower (*p* < 0.05) in Cu-supplemented pigs compared to control pigs but was not affected by the intestinal section or treatment x section. The duodenal mRNA expression of metallothionein1a (*MT1a*) was greater (*p* < 0.05) in Cu-supplemented pigs and was greater in CuSO_4_ than TBCC-fed pigs. These data demonstrate that both the Cu level and source affect the Cu uptake and mRNA expression of Cu regulatory proteins throughout the small intestine of weanling pigs.

## Simple Summary

Copper (Cu) is an essential trace mineral required in the diet of weanling pigs with a 5 to 6 mg Cu/kg diet. However, supplementing Cu at concentrations (100 to 250 mg Cu/kg) well above their requirement is known to stimulate growth and feed intake in weanling pigs. It is unclear how the young pig regulates a high dietary Cu intake to prevent toxicity. We examined the small intestinal gene expression of Cu regulatory proteins and the distribution of Cu in weanling pigs fed Cu at their requirement or at high levels (225 mg Cu/kg). Two Cu sources differing in their water solubility were compared. The supplementation of high dietary Cu reduced the gene expression of the major transporter involved in Cu uptake in the small intestine and increased the duodenal gene expression of a Cu-binding protein that reduces Cu absorption. Copper supplementation increased the soluble and mucosal Cu concentrations in the duodenum, jejunum, and ileum. Pigs supplemented with the water-soluble Cu source had greater duodenal soluble and mucosal Cu concentrations, and a greater gene expression of Cu-binding protein than pigs fed the low water-soluble source. The results of this study enhance our understanding of how the young pig regulates a high Cu intake in the small intestine to prevent toxicity.

## 1. Introduction

The copper (Cu) requirement for the young pig is 5 to 6 mg/kg Cu [1]. However, it has been well-documented that, when weanling pigs are fed Cu (100 to 250 mg/kg diet) well above their requirement, a pharmacological response is often observed where growth and feed intake are increased [2,3]. Although several physiological changes have been observed in response to a high dietary Cu [2], the definitive mechanism(s) responsible for this pharmacological effect remains unknown. While pigs are tolerant of a high dietary Cu, it is unknown how the young pig regulates a high dietary Cu intake to prevent toxicity. The duodenum and the proximal jejunum are the primary sites for Cu absorption [4] and, thus, the small intestine serves as a key regulatory organ in Cu acquisition and utilization, as the absorption of Cu is affected by the level of dietary Cu intake [5]. During absorption and metabolism, Cu ions are actively oxidized and reduced, which can cause oxidative damage [4]. Because ionic Cu acts as a prooxidant, it is important that intracellular Cu ions be tightly regulated. It has been demonstrated in rodents that intracellular Cu is regulated by transporters, chaperones, and chelators that are responsible for the acquisition, distribution, and excretion of Cu [6,7]. Copper transporter 1 (*CTR1)* is the major transporter responsible for Cu uptake by enterocytes and other cells. Copper ATPase7a (*ATP7a*) is involved in actively transporting Cu to cuproenzymes secreted from the Golgi network into the plasma membrane and exporting Cu from the small intestine. Copper ATPase7b (*ATP7b*) is important in the secretion of Cu from the liver via biliary excretion and incorporation into ceruloplasmin. Chaperone proteins are involved in trafficking Cu to specific destinations within the cell. In the cytosol, Cu is delivered to the superoxide dismutase via the copper chaperone protein (*CCS*). Copper is transported to the cytochrome c oxidase in the mitochondria by the cytochrome c oxidase assembly protein (*COX17)*. The Cu chaperone antioxidant 1 (*ATOX1*) transports Cu to the Cu ATPase in the trans-Golgi network.

Two inorganic sources of Cu that are widely used in swine diets as growth promotants are Cu sulfate (CuSO_4_) and tribasic Cu chloride (TBCC; Cu_2_(OH)_3_Cl). These sources differ greatly in their in vitro water and acid solubility. Copper sulfate is very water-soluble (99%), whereas very little (<1%) of Cu from TBCC is soluble in water [8]. The gene expressions of proteins involved in Cu metabolism have been characterized in the liver of weanling pigs fed high dietary Cu [9,10], but limited research has evaluated expression of proteins involved in regulating Cu metabolism in the small intestine of weanling pigs. Huang et al. [11] assessed the effect of the dietary Cu level and source on Cu transporter and chaperone proteins in the small intestine of weanling pigs fed a complex diet for 11 days. Newly weaned pigs are generally fed phase diets for 28 to 35 days with the final phase diet consisting largely of corn and soybean meal. The present study was conducted with weanling pigs to determine the effects of the level and source of dietary Cu on digesta soluble Cu and mucosal Cu concentrations, and the mRNA expression of proteins involved in regulating the Cu metabolism in different sections of the small intestine after a 35-day phase feeding period. Mucosal iron (Fe) and zinc (Zn) concentrations were also measured since they interact with Cu [12]. Our hypothesis is that the Cu concentration and source will alter the soluble and mucosal Cu concentrations and the gene expression of proteins involved in the regulation of the Cu metabolism in the small intestine. The performance, oxidative stress markers, and intestinal morphology results from this study were published previously [9].

## 2. Materials and Methods

### 2.1. Animals and Experimental Design

Prior to initiation of the study, animal care, handling, and sampling procedures were approved by the North Carolina State University Animal Care and Use Committee. Thirty weanling Large White x Duroc barrows (castrated males; mean weight = 6.34 kg; range = 5.00 to 7.59 kg) were weaned at approximately 21 days of age. Pigs were blocked by litter of origin and initial body weight (BW) using a randomized complete block design. Within each litter block, barrows were ranked by BW and paired into light, medium, and heavy weight categories. Each weight-paired duo of littermates was assigned to one of three pens, with 2 pigs per pen. Within each litter block, pens were randomly assigned to one of three dietary treatments: (1) control (analyzed mean of 6.7 mg Cu/kg diet), (2) 225 mg supplemental Cu/kg from CuSO_4_, or (3) 225 mg supplemental Cu/kg from TBCC. This design provided 15 pens total and 5 replications (litter block, rep) per treatment, with each treatment represented once within each litter block. Pens provided ad libitum access to water and feed. To transition pigs from their mother’s milk to a solid diet, diets were fed in three phases (Table 1). Phase 1 diet was a complex formulated diet containing milk proteins and was fed from day 0 to 6. The phase 2 diets, decreasing in milk proteins, were fed from day 7 to 20. Phase 3 diets were fed from day 21 until harvest on day 35 or 36 when pigs were 56 to 57 days of age. Diets were formulated to meet or exceed NRC requirements [1]. The only difference in diets within each phase was the level of supplemental Cu and diets were similar in protein and energy. Calculated levels of nutrients in diets are shown in Table 2.

### 2.2. Sample Collection and Analytical Procedures

Prior to harvest on day 35 or 36, pigs were fasted for 8 h, then re-fed for 8 h. Pigs were fasted and re-fed so that all pigs would be in an equal fed state. Three pens representing all dietary treatments were euthanized via captive bolt within a specific time frame on each day. As each pig was euthanized, the abdominal cavity was lacerated vertically for sample collection. To prevent the flow of digesta contents out of the intestine, the ileal-cecal junction and proximal duodenum were quickly ligated with umbilical tape with minimal movement of the gastrointestinal tract to prevent unwanted flow of digesta contents within the tract. The duodenum represented the first 60 cm distal to the pyloric valve. Approximately 152 cm from the distal end of the duodenum was measured and, at that point, a 60 cm section was obtained to represent the proximal jejunum. The 60 cm proximal to the ileal–cecal junction represented the ileum. As each intestinal section was removed, digesta contents were collected into a 50 mL conical tube and pH measurements were immediately recorded. The digesta contents were then placed on ice for determination of soluble Cu concentrations. Each section of the intestine was then opened and rinsed with 1 X PBS (Sigma-Aldrich, St. Louis, MO, USA) to clean the intestine epithelium of digesta and other debris. Subsequently, chilled microscope slides were used to obtain two sequential scrapings of mucosal tissue from each section. One scraping of mucosal tissue was placed into a 50 mL conical tube containing RNAlater (Ambion, Austin, TX, USA) as per manufacturer’s instructions, while the other was placed on ice in a 15 mL conical tube for mineral analysis. All pigs (10/treatment; 2 pigs/pen) were used for gene expression and mineral analysis.

### 2.3. Digesta and Mucosal Mineral Analysis

Immediately following sample collection, digesta samples were brought to the laboratory and processed for subsequent determination of soluble Cu concentrations. From each section, digesta samples were filtered through cheesecloth to separate the solid and liquid fractions. The liquid fraction was then centrifuged at 15,000× *g* for 10 min to separate out any remaining solid particles and then frozen at −20 °C until Cu determination.

Approximately 1 g of mucosal scrapes from the duodenum, proximal jejunum, and ileum were dried in a forced-air oven (60 °C: Imperial II; Lab-Line Instruments, Thermo Scientific, Dubuque, IA, USA). Mucosal and feed samples were prepared for mineral analysis by wet ashing using a Microwave (Mars 5; CEM Corp, Matthews, NC, USA) procedure described previously [13]. Mineral analysis was conducted via atomic absorption spectrophometry (AA-6701F; Shimadzu, Kyoto, Japan) and a NIST certified liver standard (SRM#1577c, Gaithersburg, MD, USA was included in the analysis to validate instrument accuracy. Supernatant from digesta samples was aspirated directly into the flame.

### 2.4. RNA Isolation and Quantitative Real-Time PCR

Total RNA was isolated from the duodenum, proximal jejunum, and ileum via the Qiagen RNeasy kit with an on-column DNase digestion (Qiagen, Valecia, CA, USA). Quantity and integrity of RNA was determined via NanoDrop-1000 spectrophotometer (Thero Scientific, Dubuque, IA, USA) and integrity of 28s and 16s ribosomal subunits was captured on a 1.2% agarose gel. Subsequently, 1 µg of total RNA was used to synthesize cDNA using the High-Capacity cDNA Reverse-Transcription Kit (Applied Biosystems, Inc., Foster City, CA, USA) as per manufacturer’s instructions. Quantitative real-time PCR was run in duplicate (Bio-Rad, Hercules, CA, USA) and each reaction contained 50 ng cDNA, 10 µM each of forward and reverse primers, and 1X SYBR Green Master Mix (Ambion, Auston, TX, USA). The cycling program was reported previously [14]. Genes targeted in the intestinal sections included *CTR1*, *ATP7a*, *ATP7b*, *CCS*, *COX 17*, *ATOX 1*, Cu/Zn superoxide dismutase (*SOD1*), divalent metal transporter 1 (*DMT1*), and hephaestin (*HEPH*). Real-time primers were designed with Beacon Designer software version 8.1 (Premier Biosoft Intl., Palo, CA, USA) to be compatible with SYBR Green I (Table 3) as described by [14]. One amplicon from each reaction was sequenced and validated to confirm primer specificity. Primer specificity was also confirmed by melt curve analysis and determination of product size via agarose gel electrophoresis. Numerous housekeeping genes, taken from Nygard et al. [15], were assessed for their stability in each section of the intestine. Of those assessed, *Rpl4*, *Tbp*, *Hprt1*, and *β-actin* were found to be the most stable in all sections. Within each section, the geometric mean of the housekeeping genes was determined for target normalization. Relative expression of target genes was determined via the 2^−∆∆CT^ method [16].

### 2.5. Statistical Analysis

Data were analyzed as a randomized complete block design using a linear mixed model with the MIXED procedure in SAS version 9.4 [17]. The individual pen (n = 15) served as the true experimental unit for all parameters. For gene expression and tissue mineral analyses, individual values for the two pigs housed within the same pen were averaged prior to statistical analysis to yield a single, consolidated pen-level observation. Fixed effects included treatment, intestinal section, and their interaction. Random effects included harvest block (n = 2) and rep (litter block; n = 5). Consequently, maternal and genetic litter-related effects were completely absorbed and accounted for within the random effect of the litter replicate block in the mixed model. Because of the small sample size, degrees of freedom for fixed effects were estimated using the Kenward–Roger adjustment. When the treatment × section interaction was significant, data were analyzed by individual intestinal section. Model residuals and random effects were evaluated visually for normality and homogeneity of variance using PROC UNIVARIATE to review Q-Q plots and residual histograms generated with ODS Graphics. Pairwise comparisons among treatment least squares means were performed using the pdiff option. The standard errors of the means (SEMs) reported in the tables represent the pooled SEM derived from the residual variance of the mixed model. Statistical significance was declared at *p* ≤ 0.05.

## 3. Results

### 3.1. Intestinal pH and Soluble Cu and Mucosal Cu, Fe, and Zn Concentrations

Intestinal pH was affected by section (*p* = 0.01) but not by treatment or treatment x section (Table 4). The pH was lower (*p* < 0.05) in the duodenum than in the jejunum and ileum (Table 5). Ileal pH was also lower (*p* < 0.05) than in the proximal jejunum.

Soluble Cu was affected (*p* = 0.01) by treatment, section, and treatment x section (Table 4). Control pigs had markedly lower (*p* < 0.05) soluble Cu concentrations in all intestinal sections than those supplemented with Cu. Soluble Cu concentrations in the duodenum were greater (*p* < 0.05) in pigs supplemented with CuSO_4_ compared to those fed TBCC. Pigs fed the two Cu sources had similar soluble Cu concentrations in the proximal jejunum and ileum. The duodenal soluble Cu concentrations were much lower (*p* < 0.05) than in the jejunum and ileum (Table 5). Mucosal Cu concentrations were also affected (*p* = 0.01) by treatment, section, and treatment x section (Table 4). Control pigs had lower (*p* < 0.05) mucosal Cu levels in all sections compared with those receiving supplemental Cu. Pigs supplemented with CuSO_4_ had greater (*p* < 0.05) mucosal Cu concentrations in the duodenum than those fed TBCC. However, in the proximal jejunum and ileum, mucosal Cu concentrations were greater (*p* < 0.05) in pigs supplemented with TBCC compared to those receiving CuSO_4_. Mucosal Cu was greater (*p* < 0.05) in the duodenum than in the proximal jejunum and ileum (Table 5).

Mucosal Fe concentrations were affected by treatment (*p* = 0.05) section (*p* = 0.01), and treatment x section (*p* = 0.02; Table 4). In the duodenum, mucosal Fe concentrations were greater (*p* < 0.05) in pigs supplemented with CuSO_4_ compared with those fed TBCC. Mucosal Fe did not differ amount treatments in the jejunum and ileum. The duodenal mucosal Fe concentrations were much greater (*p* < 0.05) than those in the jejunum and ileum (Table 5). Mucosal Zn concentrations were affected by intestinal section (*p* = 0.01) but not be treatment or treatment x section. Mucosal zinc concentrations were greater (*p* < 0.05) in the duodenum than in the jejunum and ileum.

### 3.2. mRNA Gene Expression of Cu Regulatory Proteins in the Small Intestine

The small intestinal mRNA expression of Cu regulatory proteins is presented in Table 6. The intestinal mRNA expression of *CTR1* was higher (*p* < 0.05) in controls than in pigs supplemented with CuSO_4_ or TBCC. The *Metallothionein* mRNA expression was affected (*p* = 0.01) by treatment, section, and treatment x section. In the duodenum, the *MT1a* mRNA expression was greater (*p* < 0.05) in Cu-supplemented pigs compared to control pigs and was greater (*p* < 0.05) in the duodenum of pigs receiving CuSO_4_ than in those fed TBCC. The *Metallothionein* expression was much greater (*p* < 0.05) in the duodenum than in the proximal jejunum and ileum (Table 5). The expression of other Cu regulatory proteins was not affected by dietary Cu or intestinal section.

## 4. Discussion

In the present study, we supplemented 225 mg Cu/kg diet from two inorganic sources of Cu whose in vitro water and acid solubility differ to examine how the level and source affects the Cu metabolism in the small intestine of weanling pigs. As expected, the Cu level and source affected intestinal soluble and mucosal Cu concentrations. Soluble and mucosal Cu concentrations were greater for CuSO_4_- compared to TBCC-fed pigs in the duodenum. In younger pigs fed a high dietary Cu for a shorter period, CuSO_4_-fed pigs had a higher mucosal Cu than those receiving TBCC [11]. However, the soluble Cu did not differ among Cu sources in this study. The soluble Cu in the jejunum and ileum did not differ among Cu sources, suggesting that the Cu from TBCC did become soluble further down the small intestine. In contrast to the duodenal results, the mucosal Cu concentrations in the proximal jejunum and ileum were greater for TBCC compared to CuSO_4_ pigs. Since Cu is absorbed primarily from the duodenum and proximal jejunum, this suggests that pigs fed TBCC absorbed more Cu from the proximal jejunum than those receiving CuSO_4_. The absorption of a mineral is determined by the extent to which the mineral becomes soluble in the stomach and the extent of ligand(s) and mineral interactions that occur during digestion [18]. The copper from TBCC may not become completely solubilized in the stomach and may gradually become soluble as the digest descends down the tract. Even in 0.1% HCl at a pH of 2.22, TBCC was less soluble and became soluble at a slower rate than CuSO_4_ [8]. Tribasic copper chloride was also less soluble than CuSO_4_ when incubated in a 0.2 mM glycine-HCL buffer at pH 2.5 for 1 h [19]. Another possibility is that these Cu sources may interact differently with exogenous ligands such as phytate. In the presence of phytate in vitro, the amount of insoluble Cu-phytate complex formed was greater for CuSO_4_ than TBCC at a pH of 5.5 and 6.5 [20]. The in vitro release of phytate -*p* following the phytase addition was less for CuSO_4_ compared to TBCC when varying concentrations of Cu were added to the incubation solution [20].

The *Metallothionein 1a* mRNA expression was much greater in the duodenum of pigs fed a high Cu compared with controls. This can be explained by the much higher duodenal mucosal Cu concentrations in Cu-supplemented pigs. Copper induces *MT1a* expression and the chelation of Cu to the MT1a protein serves to protect against Cu toxicity [7]. Copper bound to MT1a can also be lost via endogenous losses such as the sloughing of enterocytes [21]. Consistent with differences in the duodenal mucosal Cu between Cu sources, the mRNA expression of *MT1a* in the duodenum was greater in CuSO_4_ pigs compared with those fed TBCC. Pigs supplemented with cuprous oxide (Cu_2_O), a poorly water-soluble source of Cu, also had a lower mRNA expression of *MT1a* in the duodenum than those receiving CuSO_4_ [22].

The greater duodenal mucosal Cu concentrations in pigs fed CuSO_4_ compared to those receiving TBCC may have affected their intestinal function. Recent studies indicate that a high dietary Cu from CuSO_4_ can alter the intestinal morphology and gut barrier function [23]. The addition of a high dietary Cu (from CuSO_4_) to control diets decreased the villus height in the duodenum [9] and jejunum [9,23] of weanling pigs. The duodenal and jejunal villus height of pigs supplemented with 225 mg Cu/kg, from TBCC did not differ from controls [9]. The duodenal malondialdehyde (MDA) concentrations were lower in control pigs than those supplemented with CuSO_4_, while the duodenal MDA concentrations did not differ among pigs fed TBCC, and control or CuSO_4_-supplemented pigs [9]. Malondialdehyde is a product of lipid peroxidation and is often used as an indicator of oxidative stress. High cellular Cu concentrations can contribute to oxidative stress through the formation of free radicals that lead to lipid peroxidation [4]. Free Cu in the cell is normally maintained at low concentrations by Cu-binding proteins such as MT and Cu chaperone proteins [7]. However, when cellular Cu concentrations overwhelm Cu homeostasis, free Cu can cause the formation of free radicals [7]. An elevated dietary Cu may also alter gut barrier function. The supplementation of 250 mg Cu/kg (from CuSO_4_) to weanling pig diets reduced the mRNA and protein expression of several tight junction proteins in the jejunum [23].

The *Copper transporter 1* mRNA expression was downregulated in the small intestine of pigs fed a high Cu. The decreased expression of *CTR1* in pigs supplemented with 225 mg Cu/kg was likely a homeostatic attempt to limit the Cu uptake in the small intestine. The major transporter responsible for the Cu uptake by intestinal cells is CTR1 [6,7]. The intestinal deletion of CTR1 in mice results in severe Cu deprivation, as well as perturbations in growth, Fe metabolism, and cardiac function [24]. Weanling female pigs supplemented with 160 mg Cu/kg had a lower duodenal mRNA expression of *CTR1* than pigs supplemented with a 80 mg Cu/kg diet [22]. However, the expression of this gene in controls receiving 15 mg added Cu/kg did not differ from those supplemented with 80 or 160 mg Cu/kg [23]. The addition of 5 or 20 mg Cu/kg to a control diet also downregulated *CTR1* mRNA in the jejunum of finishing pigs [25]. The addition of 30 or 120 µM Cu to porcine intestinal epithelial cells also downregulated the *CTR1* gene expression [26]. Increasing the Cu from concentrations of 20 to 200 µM also decreased the CTR1 gene expression in porcine jejunum organoids [27]. In finishing pigs, increasing the dietary Cu from a 48 to 209 mg/kg diet did not affect the *CTR1* mRNA expression [28].

The lower mucosal Fe concentration in pigs supplemented with TBCC compared to CuSO_4_ is consistent with the previous findings in younger pigs [11]. Liver Fe concentrations were also lower in young pigs fed TBCC than those receiving CuSO_4_ [11] and tended to be lower in those fed TBCC in the present study [9]. The mucosal and liver Fe concentrations observed in pigs supplemented with TBCC were considerably above those reported in Fe-deficient weanling pigs and were similar to those seen in pigs fed adequate Fe (120 mg Fe/kg diet) [14]. Inorganic Fe is absorbed primarily in the duodenum [29]. It is unclear why the Cu source affected the duodenal mucosal Fe. The intestinal mRNA expression of *DMT1*, the major transporter involved in the non-heme Fe uptake by the intestinal lumen, was not affected by the Cu level or source. The Cu-dependent ferroxidase hephaestin that is critical for Fe absorption was also not affected by Cu. Hephaestin oxidizes ferrous Fe^+2^ to ferric Fe^+1^ as it exits the enterocytes, allowing ferric Fe to bind to transferrin for transport throughout the body [30].

The dietary Cu level and source did not affect the intestinal mucosal Zn concentrations. In young pigs fed a complex diet for 11 days, pigs supplemented with 225 mg Cu/kg had greater mucosal Zn concentrations in the ileum than controls [11]. Liver Zn concentrations were also greater in Cu-supplemented pigs in the present study [9]. The greater mucosal Zn concentrations in the jejunum and ileum are consistent with the jejunum and ileum being the major sites of Zn absorption [31].

## 5. Conclusions

In conclusion, our findings demonstrate that the level and source of dietary Cu affect the amount of soluble Cu in the digesta and the mucosa of the small intestine. The soluble and mucosal Cu concentrations in the duodenum were greater in pigs supplemented with CuSO_4_ compared to those receiving TBCC. The mucosal Cu concentrations in the proximal jejunum and ileum of pigs supplemented with TBCC were greater than in those fed CuSO_4_. The copper source did not affect the soluble Cu concentrations in jejunum and ileum. The mucosal Fe concentrations in the duodenum were lower for TBCC- versus CuSO_4_-supplemented pigs. Pigs supplemented with 225 mg Cu/kg had a lower mRNA expression of *CTR1* than controls. The duodenal *MT1a* mRNA expression was less in control than Cu-supplemented pigs and CuSO_4_-fed pigs had a greater *MT1a* expression than TBCC-supplemented pigs. These data provide a better understanding of how the young pig regulates pharmacological levels of Cu and indicate that the Cu source affects the Cu metabolism in the small intestine.

## Figures and Tables

**Table 1:** Composition of nursery diets ^1^.

	Phase 1	Phase 2	Phase 3
	g/kg diet		
Ingredient			
Corn, ground	383.0	458.0	615.0
Soybean meal	220.0	290.0	340.0
Dried whey	275.0	175.0	---
Plasma protein	40.0	30.0	---
Fish meal	45.0	---	---
Poultry fat	20.0	20.0	20.0
Dicalcium phosphate	4.0	15.0	14.0
Vitamin mix ^2^	0.9	0.9	0.9
Limestone	7.0	7.0	7.0
Salt	4.5	3.5	2.5
Trace mineral mix ^3^	0.6	0.6	0.6

^1^ Basal diet in each phase provided (per kilogram of diet): 6.8 mg Cu, 258.8 mg Fe, 144.3 mg Zn (phase 1); 6.6 mg Cu, 288.9 mg Fe, 141.4 mg Zn (phase 2); and 6.7 mg Cu, 221.5 mg Fe, 129.1 mg Zn (phase 3). ^2^ Provided (per kilogram of diet): vitamin A 18,512 IU, vitamin D_3_ 2639 IU, vitamin E 106.0 IU, vitamin B_12_ 0.066 mg, riboflavin 13.2 mg, niacin 79.4 mg, pantothenic acid 52.9 mg, menadione 8.7 mg, folic acid 4.0 mg, and biotin 0.53 mg. ^3^ Provided (per kilogram of diet): 80 mg Fe as FeSO_4_, 0.3 mg Se as Na_2_SeO_3_, 80 mg Zn as ZnSO_4_, 25 mg Mn as MnO, and 0.5 mg I as Ca(IO_3_)_2_(H_2_O).

**Table 2:** Calculated composition of nursery diets.

	Phase 1	Phase 2	Phase 3
ME, kcal/kg	3390	3370	3400
Crude protein, %	22.4	21.5	20.5
Calcium, %	0.88	0.83	0.69
Available phosphorus, %	0.50	0.50	0.34
SID ^1^ amino acids, %			
Lysine	1.33	1.18	1.02
Threonine	0.86	0.79	0.68
Methionine	0.33	0.28	0.29
Methionine + cysteine	0.69	0.63	0.59
Tryptophan	0.26	0.25	0.23
Isoleucine	0.85	0.82	0.78
Valine	0.98	0.93	0.86
Arginine	1.20	1.25	1.31
Histidine	0.54	0.52	0.50
Leucine	1.72	1.65	1.56
Phenylalanine	0.95	0.95	0.94
Phenylalanine + tyrosine	1.66	1.66	1.63

^1^ Standardized ileal digestible.

**Table 3:** Real-time polymerase chain reaction primers.

Primer	Accession #	Primer Sequence (5′–3′) ‡	Product Length
*β-actin* †	DQ845171	F: CACGCCATCCTGCGTCTGGA R: AGCACCGTGTTGGCGTAGAG	100
*RPL4* †	DQ845176	F: CAAGAGTAACTACAACCTTC R: GAACTCTACGATGAATCTTC	122
*TBP* †	DQ845178	F: AACAGTTCAGTAGTTATGAGCCAGA R: AGATGTTCTCAAACGCTTCG	153
*HPRT1* †	DQ845175	F: GGACTTGAATCATGTTTGTG R: CAGATGTTTCCAAACTCAAC	91
*CTR1*	NM214100.2	F: ATGATGATGATGCCTATGACC R: GATGCTGACTTGGGACTTG	150
*ATOX1*	NM001167641.1	F: CCGAAGCACGAGTTCTCC R: TGTTGGGCAGGTCAATGTC	109
*COX17*	NM001190922.1	F: GAGCACTGTGGACACCTAATTGAG R: TCACAACGCAGACCACCATTTC	86
*ATP7a*	AB271958.1	F: AAGGAGGAGACAAAGACTTCATC R: CGGATTAACTCTGCTATCATCAAG	200
*ATP7b*	XM001925351.1	F: TCACTAAGAAGCCTGGAAG R: ATGGGTGCCTTTGACATC	148
*MT1a*	NM001001266.1	F: TCCTGCTCCACTGGTAAG R:AAATACACCCTCCTCCAAAC	65
*HEPH ║*	TC328481	F: GGCGCAACAGGAGGTCTATC R: GGCCTGCTGGGAGTACATTC	100
*DMT1 ║*§	GI:190360608	F: AGGATCTAGGGCATGTGGTG R: CCACAGTCCAGGAAGGACAT	124

‡ F: Forward; R: Reverse. † Taken from [15]. ║ Taken from [14]. § Contains 3′iron responsive element.

**Table 4:** Effect of dietary concentration and source of Cu on small intestinal pH and Cu, Fe, and Zn concentrations ^1^.

	Treatment						
	Control	CuSO_4_	TBCC	SEM	Trt	Section ^2^	Trt x Section
					*p*-value		
pH	6.34	6.48	6.32	0.13	0.37	0.01	0.88
Soluble Cu, mg/L	0.63	50.5	58.9	5.1	0.01	0.01	0.01
Duodenum	0.45 ^c^	11.3 ^a^	7.9 ^b^	0.68			
Proximal jejunum	0.82 ^c^	66.9 ^b^	93.7 ^a^	10.3			
Ileum	0.70 ^b^	73.4 ^a^	74.9 ^a^	11.3			
Mucosal Cu, mg/kg DM	11.7	58.8	61.5	3.0	0.01	0.01	0.01
Duodenum	15.2 ^c^	131.1 ^a^	103.5 ^b^	7.3			
Proximal jejunum	10.7 ^c^	22.2 ^b^	42.3 ^a^	5.6			
Ileum	9.6 ^c^	23.6 ^b^	38.2 ^a^	4.1			
Mucosal Fe, mg/kg DM	321.5	400.5	246.4	41.9	0.05	0.01	0.02
Duodenum	802.5 ^a,b^	1052.5 ^a^	586.7 ^b^	125.5			
Proximal jejunum	85.7	74.7	77.9	4.4			
Ileum	76.9	75.0	73.7	5.7			
Mucosal Zn, mg/kg DM	118.8	112.2	114.0	7.6	0.24	0.01	0.07

^1^ Control pigs received no supplemental Cu and those in the copper sulfate and tribasic copper chloride treatments were supplemented with 225 mg Cu/kg diet. ^2^ Significant section effects are presented in Table 5. ^a,b,c^ Means in a row not sharing a common superscript letter differ (*p* < 0.05).

**Table 5:** Effect of small intestinal section on pH, soluble Cu, mucosal Cu, Fe, and Zn concentrations, and *MT1a* mRNA.

	Small Intestine Section			
	Duodenum	Proximal Jejunum	Ileum	SE
pH	5.75 ^c^	6.84 ^a^	6.54 ^b^	0.13
Soluble Cu, mg/L	6.5 ^b^	53.8 ^a^	49.7 ^a^	5.1
Mucosal Cu, mg/kg DM	83.4 ^a^	24.8 ^b^	23.8 ^b^	3.0
Mucosal Fe, mg/kg DM	813.9 ^a^	79.4 ^b^	75.9 ^b^	41.9
Mucosal Zn, mg/kg DM	106.9 ^b^	115.4 ^a^	122.7 ^a^	7.6
*MT1a* mRNA	18.7 ^a^	2.4 ^b^	2.5 ^b^	3.7

^a,b,c^ Means in a row not sharing a common superscript letter differ (*p* < 0.05).

**Table 6:** Effect of dietary concentration and source of Cu on mRNA expression of Cu regulatory proteins in the small intestine ^1,2,3^.

	Treatment						
	Control	CuSO_4_	TBCC	SEM	Trt	Section ^4^	TrtxSection
					*p*-value		
*CTR1*	1.89 ^a^	1.01 ^b^	0.75 ^b^	0.38	0.02	0.89	0.80
*ATP 7A*	1.18	1.12	1.18	0.14	0.95	0.64	0.38
*ATP 7B*	1.09	1.06	1.10	0.11	0.97	0.70	0.34
*ATOX1*	1.15	1.70	1.31	0.24	0.25	0.35	0.18
*CCS*	0.93	1.33	1.17	0.12	0.09	0.75	0.99
*COX17*	1.07	1.15	1.13	0.11	0.87	0.91	0.57
*DMT1*	1.18	1.73	1.81	0.38	0.45	0.30	0.20
*HEPH*	1.48	1.10	1.23	0.19	0.37	0.75	0.55
*MT1a*	1.51	15.12	7.01	3.76	0.01	0.01	0.01
Duodenum	0.73 ^c^	39.9 ^a^	15.6 ^b^	8.4			
Proximal jejunum	1.85	2.28	3.22	2.25			
Ileum	1.96	3.18	2.23	1.13			
*SOD1*	0.96	1.45	1.15	0.17	0.12	0.67	0.92

^1^ Control pigs received no supplemental Cu and those in the copper sulfate and tribasic copper chloride treatments were supplemented with 225 mg Cu/kg diet. ^2^ Means are relative expression of target gene normalization to geometric mean of the housekeeping genes ribosomal protein L4 (*Rpl 4*), TATA box binding protein (*Tbp*), hypoxanthine phosphoribosyltransferase 1 (*Hprt 1)*, and beta actin (*B-actin*). ^3^ Abbreviations: *CTR 1*, Cu transporter protein; *ATP 7A*, ATPase copper-transporting alpha; *ATP 7B*, ATPase copper-transporting beta; *ATOX 1*, antioxidant protein; *CCS*, copper chaperone for superoxide dismutase; *COX 17*, cytochrome c oxidase copper chaperone; *DMT 1*, Divalent metal transporter 1; *HEPH*, hephaestin; *MTIa*, metallothionein 1a; and *SOD 1*, superoxide dismutase. ^4^ Significant section effects are presented in Table 5. ^a,b,c^ Means in a row not sharing a common superscript letter differ (*p* < 0.05).

## Data Availability

The original contributions of this study are in the paper. Contact the corresponding author for additional inquiries.

## References

[1] National Research Council (2012). Nutrient Requirements of Swine.

[2] Espinosa C.D., Stein H.H. (2021). Digestibility and Metabolism of Copper in Diets for Pigs and Influence of Dietary Copper on Growth Performance, Intestinal Health, and Overall Immune Status: A Review. J. Anim. Sci. Biotechnol..

[3] Miranda P.A.G., Remus A., Dalto D.B., Hilgemberg R., Jasluk G.B., Silva B.C.R., Lehnen C.R. (2024). A Systematic Review and Meta-Analysis of the Effects of Various Sources and Amounts of Copper on Nursery Piglets. Vet. Sci..

[4] Linder M.C., Hazegh-Azam A. (1996). Copper Biochemistry and Molecular Biology. Am. J. Clin. Nutr..

[5] Turnlund J.R., Keyes W.R., Anderson H.L., Acord L.L. (1989). Copper Absorption and Retention in Young Men at Three Levels of Dietary Copper by use of the Stable Isotope ^65^Cu. Am. J. Clin. Nutr..

[6] Kim B., Nevitt T., Thiele D.J. (2008). Mechanisms for Copper Acquisition, Distribution and Regulation. Nat. Chem. Biol..

[7] Lu F., Wang X., Xue X., Li D., Liu A., Qin S., Liu L. (2026). Multifaceted Role of Copper Homeostasis in Gut Health: From Molecular Mechanisms to Therapeutic Interventions. Cells.

[8] Spears J.W., Kegley E.B., Mullis L.A. (2004). Bioavailability of Copper From Tribasic Copper Chloride and Copper Sulfate in Growing Cattle. Anim. Feed Sci. Technol..

[9] Fry R.S., Ashwell M.S., Lloyd K.E., O’Nan A.T., Flowers W.L., Stewart K.R., Spears J.W. (2012). Amount and Source of Dietary Copper Affects Small Intestine Morphology, Duodenal Lipid Peroxidation, Hepatic Oxidative Stress, and mRNA Expression of Hepatic Copper Regulatory Proteins in Weanling Pigs. J. Anim. Sci..

[10] Sun R., Li M., Zhang T., Yang W., Yang L. (2025). Effects of Dietary Copper Sources and Levels on Liver Copper Metabolism and the Expression of Transporters in Growing Pigs. Animals.

[11] Huang Y.L., Ashwell M.S., Fry R.S., Lloyd K.E., Flowers W.L., Spears J.W. (2015). Effect of Dietary Copper Amount and Source on Copper Metabolism and Oxidative Stress of Weanling Pigs in Short-Term Feeding. J. Anim. Sci..

[12] Einhorn V., Haase H., Maares M. (2024). Interaction and Competition for Intestinal Absorption by Zinc, Iron, Copper, and Manganese at the Intestinal Mucus Layer. J. Trace Elem. Med. Biol..

[13] Gengelbach G.P., Ward J.D., Spears J.W. (1994). Effects of Dietary Copper, Iron, and Molybdenum on Growth and Copper Status of Beef Cows and Calves. J. Anim. Sci..

[14] Hansen S.L., Trakooljul N., Liu H.C., Moser A.J., Spears J.W. (2009). Iron Transporters are Differentially Regulated by Dietary Iron, and Modifications are Associated with Changes in Manganese Metabolism in Young Pigs. J. Nutr..

[15] Nygard A.B., Jorgensen C.B., Cirera S., Fredholm M. (2007). Selection of Reference Genes for Gene Expression Studies in Pig Tissues Using SYBR Green qPCR. BMC Mol. Biol..

[16] Lavak K.J., Schmittgen T.D. (2001). Analysis of Relative Gene Expression Data Using Real-Time Quantitative PCR and the 2^−ΔΔct^ Method. Methods.

[17] SAS Institute (2016). Version 9.4 of the SAS System for Windows.

[18] Powell J.J., Whitehead M.W., Lee S., Thompson R.P.H. (1994). Mechanisms of Gastrointestinal Absorption: Dietary Minerals and the Influence of Beverage Ingestion. Food Chem..

[19] Pang Y., Applegate T.J. (2007). Effects of Dietary Copper Supplementation and Copper Source on Digesta pH, Calcium, Zinc, and Copper Complex Size in the Gastrointestinal Tract of the Broiler Chicken. Poult. Sci..

[20] Pang Y., Applegate T.J. (2006). Effects of Copper Source and Concentration on in Vitro Phytate Phosphorus Hydrolysis by Phytase. J. Agric. Food Chem..

[21] Hoogenraad T.U. (2006). Paradigm Shift in Treatment of Wilson’s Disease: Zinc Therapy Now Treatment of Choice. Brain Dev..

[22] Van Baal J., Kruijt L., Binnendijk G.P., Durosoy S., Romeo A., Bikker P. (2024). Influence of Copper Source and Dietary Inclusion Level on Growth Performance of Weaned Pigs and Expression of Trace Element Related Genes in the Small Intestine. Animal.

[23] Liao J., Li Q., Lei C., Yu W., Deng J., Guo J., Han Q., Hu L., Li Y., Pan J. (2021). Toxic Effects of Copper on the Jejunum and Colon of Pigs: Mechanisms Related to Gut Barrier Dysfunction and Inflammation Influenced by the Gut Microbiota. Food Funct..

[24] Nose Y., Kim B.E., Thiele D.J. (2006). Ctr1 Drives Intestinal Copper Absorption and is Essential for Growth, Iron Metabolism, and Neonatal Cardiac Function. Cell Metab..

[25] Wen Y., Li R., Piao X., Lin G., He P. (2022). Different Copper Sources and Levels Affect Growth Performance, Copper Content, Carcass Characteristics, Intestinal Microorganisms and Metabolism of Finishing Pigs. Anim. Nutr..

[26] Li R., Wen Y., Lin G., Meng C., He P., Wang F. (2020). Different Sources of Copper Effect on Intestinal Epithelial Cell: Toxicity, Oxidative Stress, and Metabolism. Metabolites.

[27] Gao X., Liu S., Zhang S., Yang Z., Liu X., Li X., Bai Z., Deng M., Wang J., Wan D. (2026). Effects of Copper Glycinate Replacing High-Dose Copper Sulfate on Growth Performance, Trace Element Metabolism and Stem Cell Activity in Growing Pigs. Biol. Trace Elem. Res..

[28] Cobb K.F., Burnett D.D., DeRouchey J.M., Tokach M.D., Gonzalez J.M., Wu F., Dritz S.S., Goodband R.D., Woodworth J.C., Pluske J.R. (2018). Effect of Diet Type and Added Copper on Growth Performance, Carcass Characteristics, Energy Digestibility, Gut Morphology, and Mucosal mRNA Expression of Finishing Pigs. J. Anim. Sci..

[29] Miret S., Simpson R.J., McKie A.T. (2003). Physiology and Molecular Biology of Dietary Iron Absorption. Annu. Rev. Nutr..

[30] Arredondo M., Nunez M.T. (2005). Iron and Copper Metabolism. Mol. Asp. Med..

[31] Hogstrand C., Fu D., Maret W., Wedd A.G. (2014). Zinc. Binding, Transport, and Storage of Metal Ions in Biological Cells.

